# Effects of Compost-Bedded Pack Barn on Circulating Cortisol and Beta-Endorphins in Dairy Cows: A Case Study

**DOI:** 10.3390/ani11113318

**Published:** 2021-11-20

**Authors:** Rosangela Odore, Ilaria Biasato, Giulia Gardini, Antonio D’Angelo, Claudio Bellino

**Affiliations:** 1 Department of Veterinary Sciences, University of Turin, 10095 Grugliasco, TO, Italy; rosangela.odore@unito.it (R.O.); giulia.gardini@unito.it (G.G.); antonio.dangelo@unito.it (A.D.); claudio.bellino@unito.it (C.B.); 2 Department of Agricultural, Forest and Food Sciences, University of Turin, 10095 Grugliasco, TO, Italy

**Keywords:** compost barn, dairy cattle, animal welfare, cortisol, beta-endorphins

## Abstract

**Simple Summary:**

Management and housing conditions have been reported to significantly affect the health and welfare of livestock species. Therefore, the adoption of novel, alternative housing systems (such as the compost-bedded pack barn, developed in the USA) requires extensive research to assess the implications for animal welfare. From a general point of view, animal welfare is typically assessed by means of animal-based (such as blood biochemical markers) and resource-based (such as management practices) indicators. Based on such considerations, the present study evaluated the fluctuation of circulating cortisol and beta-endorphins in dairy cows housed in a conventional freestall barn (FB) and in the alternative compost-bedded pack barn (CB). The results obtained suggest that the CB housing system did not elicit significant changes in either blood cortisol or beta-endorphins.

**Abstract:**

The up-to-date literature suggests that the compost-bedded pack barn housing system is capable of remarkably improving productive and reproductive performance, as well as health status and welfare, in dairy cattle. However, there is currently limited knowledge available on the endocrine and biochemical changes in animals housed in such alternative systems. Therefore, this study aimed to measure blood cortisol (COR) and beta-endorphins (BE) in 22 two-year-old primiparae Fleckvieh cows, who were randomly allotted to the following two different housing systems: CB (*n* = 11) and FB (*n* = 11). Blood samples were collected at the beginning of the experiment (T0) and every two months thereafter (T1, T2, and T3). The COR and BE were measured through an immunoenzymatic kit. With the only exception being T0, no differences were observed over time between the two groups, neither for COR nor for BE. However, the blood cortisol levels of the CB cows decreased over time, while a T1 peak was identified in the FB group. On the contrary, both the housing systems displayed numerically higher BE at T3 than at the other experimental times. Therefore, the overall data suggest that the compost-bedded pack barn did not significantly affect the studied parameters. Accordingly, cow welfare should be assessed using a wider panel of animal-based indicators.

## 1. Introduction

In the recent decades, dairy cow welfare has become an issue of increasing concern [1,2]. Several research studies have previously quantified the economic returns of animal welfare, showing that the application of adequate standards and practices can contribute to increased food security and, in turn, farm income. On the other hand, there is growing perception among consumers that farm animal welfare should be protected and improved [3]. 

As a matter of fact, management and housing hazards may have a remarkable impact on the welfare of dairy cows, especially those linked to comfort around resting, such as bedding material type and management [4,5]. Within such a scenario, the compost-bedded pack barn (CB) is a housing system for dairy cattle that has received increasing attention in recent years [6,7,8]. Briefly, this type of barn has a large open resting area with permanent litter mixed with dry wood sawdust or other organic materials. The pack is tilled one to three times per day, thus favoring aeration and allowing the obtainment of a drier resting surface [6,7,8]. On the other hand, the compost bedding can be remarkably expensive due to the labor requirements for litter management operations, greater unitary surfaces when compared to traditional stables, and high heat production in the hot season [9,10]. Although this rearing system has been adopted for many years in the USA, it has spread to Europe later. In particular, the first Italian compost-bedded pack barn was built in 2013–2014, with high levels of satisfaction from the producers [10]. 

As welfare is the condition of the animal, animal-based measures, such as blood parameters, are also likely to provide direct information about the welfare status of the animal. In particular, cortisol (COR) is a stress steroid hormone released in response to the activation of the hypothalamic–pituitary–adrenal (HPA) axis, and it has commonly been used as a neuroendocrine biomarker of different stressful conditions in both humans and animals [11,12]. In particular, glucocorticoids have been widely used in welfare-based experimental research in farm animals, as an animal-based measure to evaluate the effects of the environment and breeding system and/or practices [13,14]. Besides the fact that conflicting research results do exist, according to Trevisi and Bertoni [15], COR can be considered a suitable candidate for chronic stress evaluation, and an increase in COR circulating levels has been observed in chronically stressed herds. In parallel, beta-endorphins (BE) are neuromodulators primarily produced by the anterior lobe of the pituitary gland, and released to the periphery in response to painful and stressful events [16]. Therefore, some research studies investigated the role of circulating BE in calves undergoing potentially stressful events [17]. 

Besides the relatively high costs, a significant improvement in productive and reproductive performance—as well as health status—has previously been reported in dairy cows housed in CB systems [6,9,18]. Moreover, observations in terms of species-specific behaviors suggest that CB systems are adequate housing systems for dairy cows [8,19]. Interestingly, Fernandez et al. [20] investigated the impact of CB on dairy cow welfare by studying some animal-based health and behavioral measures. However, there is currently limited knowledge available on the endocrine and biochemical changes in animals housed in such alternative systems. This represents a relevant issue, as the correlation between the observational/performance data and the measurements of endocrine parameters can provide useful insights into the effects of housing systems on cattle welfare.

Therefore, the aim of the present study was to measure either the blood COR or the BE levels in dairy cattle housed in a conventional freestall barn (FB) and in a CB barn. It is worth noting that, besides the fact that measuring the activation of the HPA axis represents one of the most popular approaches to study stress and well-being in animals, it is well documented that a reliable assessment of overall dairy cow welfare relies on the combination of several poor/good welfare indicators. Based on such considerations, a brief discussion of the obtained results, in light of previous data concerning clinical and behavioral findings observed in both CB and FB animals, is also provided.

## 2. Materials and Methods

The study was performed according to pending animal welfare regulations (Directive 98/58/EC and Italian Decree Law 146/2001).

### 2.1. Animals and Experimental Design

The details of the experimental design are reported by Biasato et al. [8]. Briefly, after an acclimation period of 40 days, 22 two-year-old primiparae Fleckvieh cows in early lactation were randomly allotted to the following two housing systems: FB (*n* = 11) and CB (*n* = 11).

**Figure 1:**
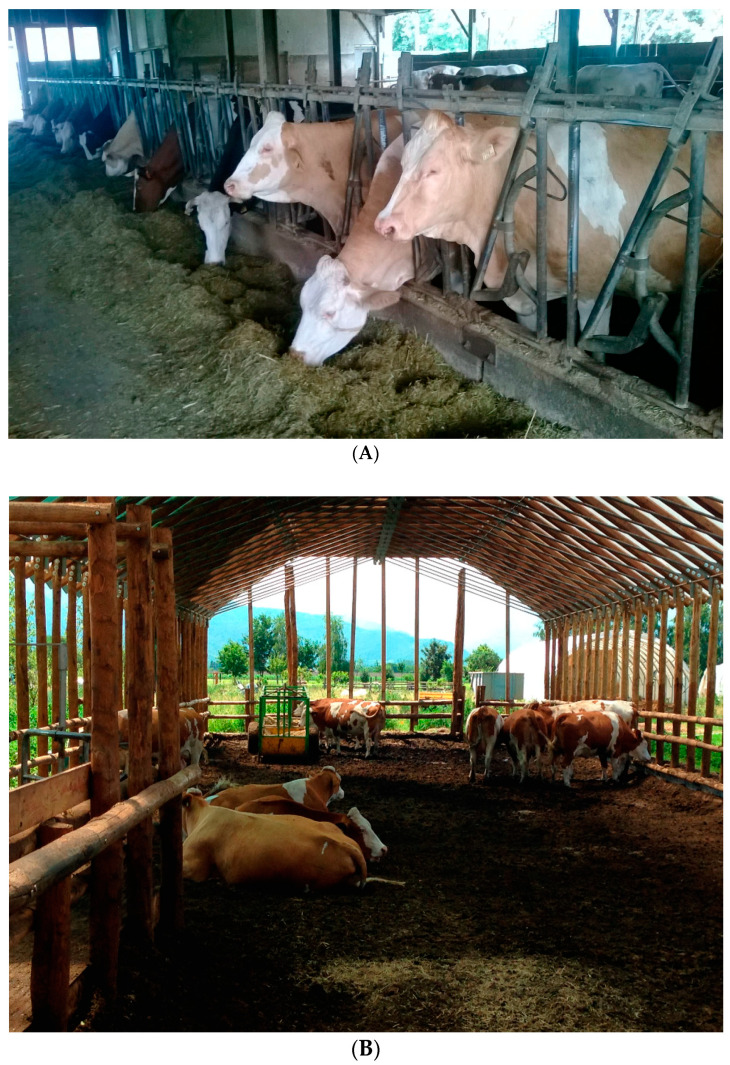
Pictures of the (**A**) freestall barn (FB) and the (**B**) compost barn (CB) housing systems.

The FB animals were provided with a space allowance of 10 m^2^/head, whereas the remaining cows (CB) were housed at a density of at least 25 m^2^/head. The conventional FB (stocking rate: 50 cows) consisted of an open-air barn arranged in a double row head-to-head housing system with a concrete floor and a central feed alley. A neck rail allowed cows to access the feed alley (Figure 1A). The resting area had divided cubicles of concrete floor lined with straw bedding. No external paddocks were present. The CB facility consisted of a large resting area open on one side, supported by wooden trusses and covered with plastic sheets. About 50 cm of permanent, organic bedding material was distributed on the floor and aerated twice daily without additional bedding added (Figure 1B). The compost used as bedding material was obtained from domestic food (60%) and vegetable (40%) waste. Its physicochemical characteristics were as follows: humidity 23.4%, pH 6.6, organic carbon (C) 35.9% of dry matter (DM), organic nitrogen (N) 95.3% of DM, carbon-to-nitrogen ratio (C:N) 19.08, and fulvic and humic acids (FA + HA) 8.48% of DM. A transportable manger with water troughs and a milking pen with places for four cows at a time were also provided, with the latter facilitating both the milking operations and the clinical examination. Before beginning the study, all the animals were tested for bovine viral diarrhoea virus (BVDV) and Mycobacterium avium subspecies paratuberculosis (MAP), and underwent a complete blood count (CBC) and biochemical profile. Animals were fed twice a day with the same quantity of total mixed ratio (also characterized by the same nutritional composition in both the housing systems) and water was provided ad libitum.

### 2.2. Blood Sampling and Analyses

All the animals had blood samples taken at T0 (at the end of the acclimation period, March 2014) and every two months thereafter (T1 May 2014, T2 July 2014 and T3 September 2014). Blood sampling was part of the farm’s health monitoring program, and an aliquot of blood was used to measure COR and BE concentrations. Blood samples were collected at the same time in the morning (between 10:00 a.m. and 11:00 a.m.) by the same expert researchers to minimize the potential, additional stress induced by sampling. 

Samples for COR concentration measurement were allowed to clot at room temperature and centrifuged (2500× *g* for 10 min at 4 °C), and aliquots of obtained sera were stored at −80 °C until assay. A commercial bovine COR (DetectX Cortisol Enzyme Immunoassay Kit, Arbor Assays, Ann Arbor, MI, USA) ELISA kit was used. As far as BE was concerned, blood was collected into EDTA tubes and centrifuged at 2000× *g* for 10 min at 4 °C. Plasma aliquots were then analyzed by means of an immunoenzymatic kit (Bovine beta-endorphin ELISA kit, Cusabio Biotech Co., Ltd., Houston, TX, USA).

### 2.3. Statistical Analysis

Considering the impossibility to replicate the housing treatment at farm level, inferential statistics could not be applied. Therefore, descriptive statistics reporting of the results as mean values ± standard deviation (SD) of triplicate measurements was used.

## 3. Results

### 3.1. Blood Cortisol

**Figure 2:**
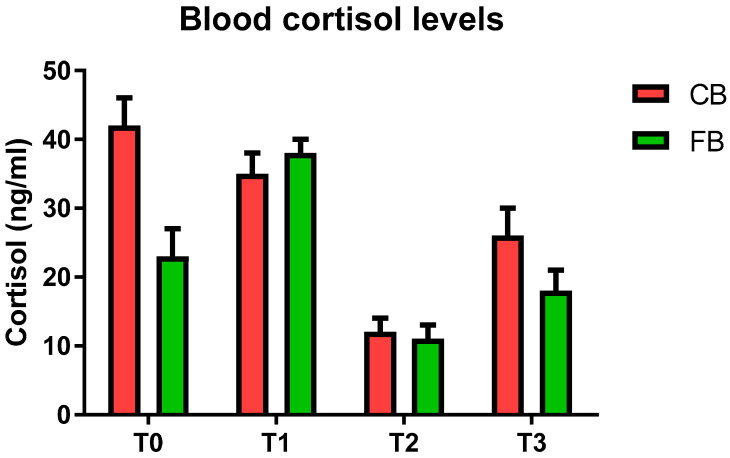
Blood cortisol levels (mean values ± SD) in the compost barn (CB) and the freestall barn (FB) groups.

At T0, the CB cows showed numerically higher blood COR levels when compared to the FB group (Figure 2). However, analogous COR levels between the CB and FB cows were detected for the other experimental times (Figure 2).

**Figure 3:**
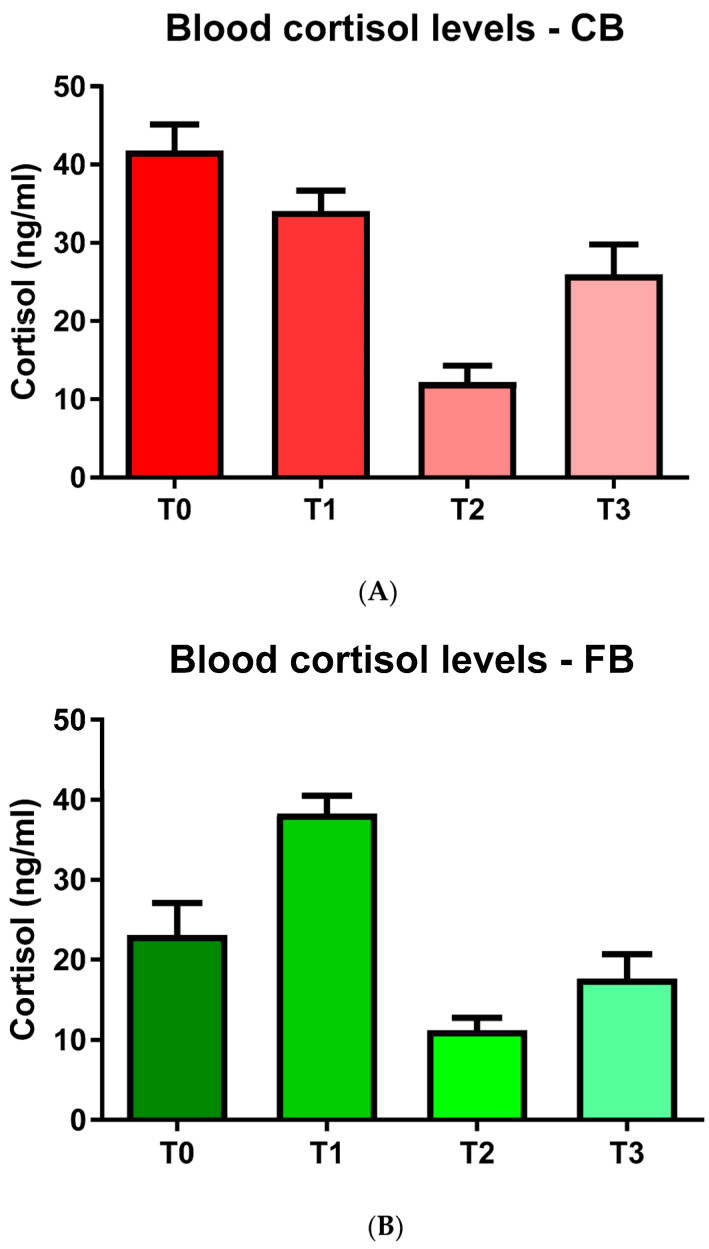
Blood cortisol levels (mean values ± SD) in the (**A**) compost barn (CB) and the (**B**) freestall barn (FB) groups.

Within the CB group, the blood COR levels decreased over time, with a more pronounced decrease observed at T2 in comparison with T0 and T1 (Figure 3A). The FB cows displayed numerically lower blood COR levels at T2 and T3 when compared to T1, respectively (Figure 3B).

### 3.2. Blood Beta-Endorphins

**Figure 4:**
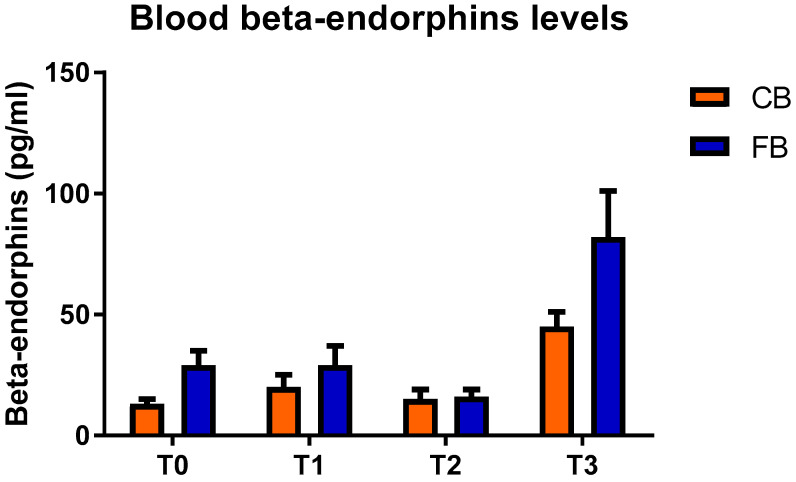
Blood beta-endorphins levels (mean values ± SD) in the compost barn (CB) and the freestall barn (FB) groups.

The BE levels were similar between the CB and FB housing systems within each experimental time (Figure 4).

**Figure 5:**
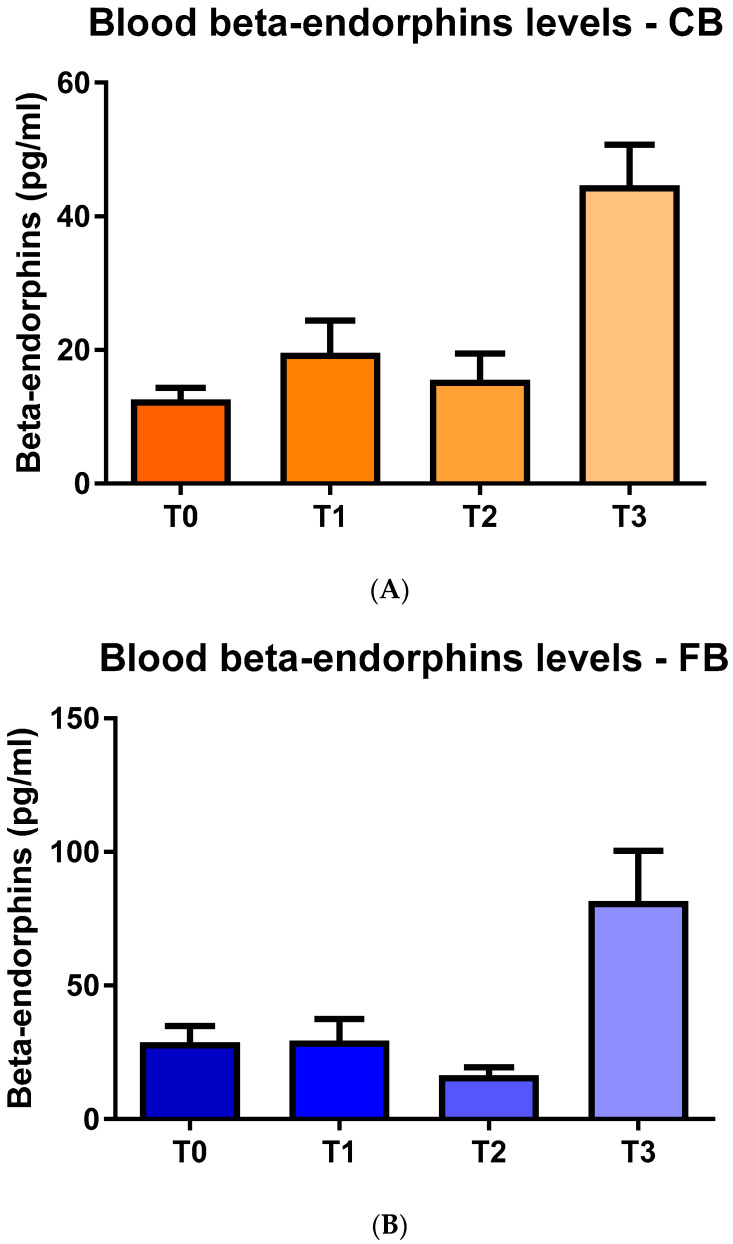
Blood beta-endorphin levels (mean values ± SD) in the (**A**) compost barn (CB) and the (**B**) freestall barn (FB) groups.

As far as the blood BE levels are concerned, in both the CB and FB groups, there was an increase at T3 when compared to the other experimental times (Figure 5A,B).

## 4. Discussion

To the best of the authors’ knowledge, this is the first study investigating blood COR and BE levels in CB-housed dairy cows. 

When comparing the two experimental groups, the obtained data overall suggest that the housing system did not significantly affect the circulating COR levels. However, while the blood COR levels tended to decrease progressively over time within the CB group, in the FB group, the decline—despite evidently occurring at T2—was irregular. Furthermore, at T0, the COR concentrations were numerically higher in the CB group when compared to the FB cows. Therefore, at T1, the COR levels were slightly decreased in the CB group. This finding may suggest an effective animal–environment interaction. It is well known that, among all the factors affecting animal welfare, density and space allowance have a key role [21,22]. Indeed, in pigs, an inverse correlation between serum COR levels and space allowances has recently been observed [23]. Gupta et al. [24] also reported that a space allowance of 1.2 m^2^, in comparison with 2.7 m^2^, is capable of eliciting the stress response in bulls, by increasing the COR levels. Considering that the CB and FB groups were provided with different space allowances, differences in circulating COR levels could have been expected. However, it is important to underline that significant increases in hematological or metabolic variables may depend on whether space allowances initiate a stress response in dairy cows. According to Kean et al. [25], increasing the space allowance above 3.0 m^2^/animal does not induce relevant modifications in the stress indicators. In the present study, the FB-housed cows were provided with a space allowance of 10 m^2^/head. Therefore, overstocking conditions were avoided in the FB group as well, with limited or no challenge at all in terms of animal welfare. Moreover, measurements were performed every two months, and this time frame could have allowed adaptation to—as well as coping with—environmental challenges. On the other hand, the duration of the study could have accounted for some influence of climate on glucocorticoid levels, especially during the summer period. It has, indeed, been demonstrated that either acute or chronic heat stress can activate the HPA axis, thus increasing peripheral COR concentrations [26]. Furthermore, heat stress in dairy cattle reduces milk production and milk quality. From this point of view, earlier results by Biasato et al. [8] suggest that environmental conditions did not challenge either the CB- or FB-housed cattle. A possible explanation for this is that the combination of different climate factors (e.g., relative humidity, solar radiation, and wind speed), rather than heat alone, may influence the stress response. 

In the present study, in both the CB and FB groups, an increase in the BE levels was detected at T3. Furthermore, consistent with the findings reported by Micera et al. [27], the circulating BE showed great individual variability. It is well recognized that these opioid peptides are synthesized by the pro-opiomelanocortin gene in response to environmental stressors, and are implicated in behavioral responses associated with these stimuli [28]. As a confirmation of such aspects, transgenic mice with low ability to synthesize BE have been reported to show a stronger aversion to novel environments by developing anxious behavior after stress exposure [29]. Limited data are currently available concerning stress-induced modifications of BE levels in domestic animals. In sport horses, a significant increase in circulating BE levels has been observed following exercise [30] In sheep, transient increases can also be identified in response to common management practices [31]. As far as cattle are concerned, the main findings deal with changes in BE levels according to the reproductive status [32]. Indeed, Aurich et al. [33] found that, in cattle, the highest BE values are reached in the last month of gestation, and they are probably related to other pregnancy-associated endocrine changes. In the present study, the majority of the animals belonging to the FB group were closer to calving, whereas the cows in the CB group had just delivered their calves (data not shown). Therefore, the increase in BE observed at T3 could be partially related to the reproductive phase and/or pregnancy. 

As a final aspect to consider, the assessment of cow well-being is a complex matter, as the responses involve behavioral changes, activation of the neuroendocrine system, and determination of productive and reproductive performance [34,35]. Either the FB- or CB-housed cows were visited every two weeks throughout the experimental period, and behavior measurements were recorded before the clinical procedures. Milk samples were also collected every month, and analyzed for the nutritional composition and the somatic cell count. Cows belonging to the CB group were comfortable and able to express normal lying behaviors, as well as exhibiting less agonistic interactions when compared to the FB-housed cows. Furthermore, the scores for hind limb cleanliness and locomotion were improved in the CB group, and the milk somatic cell count was also decreased [8]. The descriptive results reported in the present study, taken together with the findings from Biasato et al. [8], provide supporting evidence that the CB housing system does not represent a challenge to cow welfare, but that it can be considered animal friendly.

The present study has, however, some limitations. The first is undoubtedly the limited sample size, and the individual variability in COR and BE concentrations. Furthermore, a COR measurement in different biological matrices would have provided useful information to interpret hormonal fluctuations. Indeed, fecal and hair COR determination have become increasingly popular as non-invasive alternatives to monitor and quantify chronic stress in dairy cows [36,37]. However, COR detection and interpretation in other biological samples has been argued due to the lower hormone concentrations in some matrices and/or the presence of conjugated forms [38]. In particular, it has been postulated that salivary COR is poorly correlated with blood concentrations in chronic stress occurrence [15]. As a consequence, at the time of the experimental trial, COR determination in the blood remained the most suitable physiological indicator. Finally, the choice of blood as the matrix was related to practical issues, as blood sampling was already part of the farm’s health monitoring program.

## 5. Conclusions

In conclusion, neither the CB nor the FB housing systems came to elicit significant changes in blood COR and BE levels. The limited sample size, as well as the individual variability in blood concentrations, may have determined the lack of differences between the two housing systems, thus making further studies involving a wider animal population mandatory. Furthermore, considering the recent shift towards alternative methods in assessing the COR level changes in dairy cows, the choice of COR determination on different matrices (such as feces or hair) is herein recommended.

## Data Availability

The data presented in this study are available on request from the corresponding author. The data are not publicly available due to matter of farm privacy.

## References

[1] Logue D.N., Mayne C.S. (2014). Welfare-positive management and nutrition for the dairy herd: A European perspective. Vet. J..

[2] Molina L., Agüera E., Maroto-Molina F., Pérez-Marín C.C. (2019). Assessment of on-farm welfare for dairy cattle in southern Spain and its effects on reproductive parameters. J. Dairy Res..

[3] Alonso A., Gonzalez-MontanaM J.R., Lomillos J.M. (2020). Consumers’ concerns and perceptions of farm animal welfare. Animals.

[4] Cook N.B. (2003). Prevalence of lameness among dairy cattle in Wisconsin as a function of housing type and stall surface. J. Am. Vet. Med. Assoc..

[5] Bertocchi L., Fusi F., Angelucci A., Bolzoni L., Pongolini S., Strano R.M., Ginestreti J., Riuzzi G., Moroni P., Lorenzi V. (2018). Characterization of hazards, welfare promoters and animal-based measures for the welfare assessment of dairy cows: Elicitation of expert opinion. Prev. Vet. Med..

[6] Barberg A.E., Endres M.I., Salfer J.A., Reneau J.K. (2007). Performance and welfare of dairy cows in an alternative housing system in Minnesota. J. Dairy Sci..

[7] Lobeck K.M., Endres M.I., Shane E.M., Godden S.M., Fetrow J. (2011). Animal welfare in cross-ventilated, compost-bedded pack, and naturally ventilated dairy barns in the upper Midwest. J. Dairy Sci..

[8] Biasato I., D’Angelo A., Bertone I., Odore R., Bellino C. (2019). Compost bedded-pack barn as an alternative housing system for dairy cattle in Italy: Effects on animal health and welfare and milk and milk product quality. Ital. J. Anim. Sci..

[9] Leso L., Uberti M., Morshed W., Barbari M. (2013). A survey of Italian compost dairy barns. J. Agric. Eng..

[10] Leso L., Barbari M., Lopes M.A., Damasceno F.A., Galama P., Taraba J.L., Kuipers A. (2020). Invited review: Compost-bedded pack barns for dairy cows. J. Dairy Sci..

[11] Russell G., Lightman S. (2019). The human stress response. Nat. Rev. Endocrinol..

[12] Brown E.J., Vosloo A. (2017). The involvement of the hypothalamopituitary-adrenocortical axis in stress physiology and its significance in the assessment of animal welfare in cattle. Onderstepoort J. Vet. Res..

[13] Fordham D.P., Al-Gahtani S., Durotoye L.A., Rodway R.G. (2010). Changes in plasma cortisol and β-endorphin concentrations and behaviour in sheep subjected to a change of environment. Anim. Sci..

[14] Odore R., Badino P., Re G., Barbero R., Cuniberti B., D’Angelo A., Girardi C., Fraccaro E., Tarantola M. (2011). Effects of housing and short-term transportation on hormone and lymphocyte receptor concentrations in beef cattle. Res. Vet. Sci..

[15] Trevisi E., Bertoni G. (2009). Some physiological and biochemical methods for acute and chronic stress evaluation in dairy cows. Ital. J. Anim. Sci..

[16] Pilozzi A., Carro C., Huang X. (2021). Roles of β-endorphin in stress, behavior, neuroinflammation, and brain energy metabolism. Int. J. Mol. Sci..

[17] Cooper C., Evans A.C.O., Cook S., Rawlings N.C. (1995). Cortisol, progesterone and β-endorphin response to stress in calves. Can. J. Anim. Sci..

[18] Ofner-Schröck E., Zähner M., Huber G., Guldimann K., Guggenberger T., Gasteiner J. Kompoststall-funktionell und tiergerecht?. Proceedings of the Bautagung Raumberg-Gumpenstein, AREC Raumberg-Gumpenstein.

[19] Endres M.I., Bamberg A.E. (2007). Behavior of dairy cows in an alternative bedded-pack housing system. J. Dairy Sci..

[20] Fernandez A., Mainau E., Manteca X., Siurana A., Castillejos L. (2020). Impacts of Compost Bedded Pack Barns on the Welfare and Comfort of Dairy Cows. Animals.

[21] Bova T.L., Chiavaccini L., Cline G.F., Hart C.G., Matheny K., Muth A.M., Voelz B.E., Kesler D., Memili E. (2014). Environmental stressors influencing hormones and system physiology in cattle. Reprod. Biol. Endocrinol..

[22] Park R.M., Foster M., Daigle C.L. (2020). The impact of housing systems and environmental features on beef cattle welfare. Animals.

[23] Kim K.H., Kim K.S., Kim J.E., Kim D.W., Seol K.H., Lee S.H., Chae B.J., Kim Y.H. (2017). The effect of space allowance on growth performance and physiological responses of pigs at different stages of growth. Animal.

[24] Gupta S., Earley B., Crowe M.A. (2007). Pituitary, adrenal and immune performance responses of mature Holstein x Friesian bulls housed on slatted floors at various space allowances. Vet. J..

[25] Keane M.P., McGee M., O’Riordan E.G., Kelly A.K., Earley B. (2017). Effect of space allowance and floor type on performance, welfare and physiological measurements of finishing beef heifers. Animal.

[26] Bagath M., Krishnan G., Devaraj C., Rashamol V.P., Pragna P., Lees A.M., Sejian V. (2019). The impact of heat stress on the immune system in dairy cattle: A review. Res. Vet. Sci..

[27] Micera E., Dimatteo S., Grimaldi M., Marsico G., Zarrilli A. (2007). Stress indicators in steers at slaughtering. Ital. J. Anim. Sci..

[28] Grisel J.E., Bartels J.L., Allen S.A., Turgeon V.L. (2008). Influence of β-endorphin on anxious behavior in mice: Interaction with EtOH. Psychopharmacology.

[29] Barfield E.T., Moser V.A., Hand A., Grisel J.E. (2013). Beta-endorphin modulates the effect of stress on novelty-suppressed feeding. Front. Behav. Neurosci..

[30] Pazzola M., Pira E., Sedda G., Vacca G.M., Cocco R., Sechi S., Bonelli P., Nicolussi P. (2015). Responses of haematological parameters, beta-endorphin, cortisol, reactive oxygen metabolites, and biological antioxidant potential in horses participating in a traditional tournament. J. Anim. Sci..

[31] Hall S., Broom D., Goode J., Lloyd D., Parrott R., Rodway R. (1999). Physiological responses of sheep during long road journeys involving ferry crossings. Anim. Sci..

[32] Hydbring E., Madej A., MacDonald E., Drugge-Boholm G., Berglund B., Olsson K. (1999). Hormonal changes during parturition in heifers and goats related to the phases and severity of labour. J. Endocrinol..

[33] Aurich J.E., Dobrinski I., Hoppen H.O., Grunert E. (1990). Beta-Endorphin and met-enkephalin in plasma of cattle during pregnancy, parturition and the neonatal period. J. Reprod. Fert..

[34] von Borell E.H. (2001). The biology of stress and its application to livestock housing and transportation assessment. J. Anim. Sci..

[35] Biswal O., Sogamond A. (2020). Assessment of welfare through behavioural, physiological and biochemical measures in dairy animals: A review. Int. J. Livestock Res..

[36] Rees A., Fischer-Tenhagen C., Heuwieser W. (2016). Cortisol Metabolites in Dairy Cows. Reprod. Domest. Anim..

[37] Vesel U., Pavič T., Ježek J., Snoj T., Starič J. (2020). Welfare assessment in dairy cows using hair cortisol as a part of monitoring protocols. J. Dairy Res..

[38] Barrell G.K. (2019). An Appraisal of Methods for Measuring Welfare of Grazing Ruminants. Front. Vet. Sci..

